# Benefits and Challenges of Transition from High-Dose Methadone to Buprenorphine Depot: A Case Report

**DOI:** 10.1192/j.eurpsy.2025.944

**Published:** 2025-08-26

**Authors:** M. Delic

**Affiliations:** 1 Center for Treatment of Drug Addiction, University Psychiatric Clinic Ljubljana, Ljubljana, Slovenia

## Abstract

**Introduction:**

Opioid dependence is a complex condition that often requires long-term treatment and care. Methadone, a synthetic full opioid agonist, and buprenorphine, a partial agonist at the opioid receptor, are commonly used in substitution therapy for opioid dependence, typically administered as an oral liquid or sublingual tablet. Transitioning from high-dose methadone to buprenorphine for the treatment of opioid use disorder (OUD) poses a risk of precipitated withdrawal. This risk arises from introducing a high-affinity partial agonist (buprenorphine) at the mu-opioid receptor after it has been occupied by a lower-affinity full agonist (methadone). As a result, this transition is usually only performed for patients on low doses of methadone (<30-40 mg). Microdose induction has been proposed as a potential solution to facilitate a smoother transition to buprenorphine.

**Objectives:**

To present a case report of a rapid transition from high-dose methadone to buprenorphine depot, highlighting both the benefits and challenges of this process.

**Methods:**

This case report describes a patient who was switched from 150 mg of methadone to 32 mg of sublingual buprenorphine using microdosing, and subsequently transitioned to a weekly 160 mg buprenorphine depot injection.

**Results:**

The patient was successfully transitioned to sublingual buprenorphine and later to buprenorphine depot without experiencing withdrawal symptoms. Even later, the patient reported no signs of withdrawal and was satisfied with the buprenorphine dosage. The patient attended monthly check-ups with the doctor; however, 15 days after the transition, he began consuming alcohol and soon after, started using cocaine.

**Conclusions:**

This report supports the use of microdose induction for initiating buprenorphine, particularly for patients stabilized on high doses of methadone who may struggle with traditional buprenorphine induction methods. Although the patient remained abstinent from opioids, he quickly relapsed with alcohol and cocaine, issues that had not been present during his methadone treatment. Regular and more frequent therapeutic assessments are very important for many patients to prevent relapse.

**Disclosure of Interest:**

None Declared

